# Learning from –omics strategies applied to uncover *Haemophilus influenzae* host-pathogen interactions: Current status and perspectives

**DOI:** 10.1016/j.csbj.2021.05.026

**Published:** 2021-05-15

**Authors:** Nahikari López-López, Celia Gil-Campillo, Roberto Díez-Martínez, Junkal Garmendia

**Affiliations:** a Instituto de Agrobiotecnología, Consejo Superior de Investigaciones Científicas (IdAB-CSIC)-Gobierno de Navarra, Mutilva, Spain; b Telum Therapeutics, Noain, Spain; c Centro de Investigación Biomédica en Red de Enfermedades Respiratorias (CIBERES), Madrid, Spain

**Keywords:** *Haemophilus influenzae*, Airway infection, Genome, Transcriptome, Methylome, Proteome, Metabolome, Tn-seq

## Abstract

*Haemophilus influenzae* has contributed to key bacterial genome sequencing hallmarks, as being not only the first bacterium to be genome-sequenced, but also starring the first genome-wide analysis of chromosomes directly transformed with DNA from a divergent genotype, and pioneering Tn-seq methodologies. Over the years, the phenomenal and constantly evolving development of –omic technologies applied to a whole range of biological questions of clinical relevance in the *H. influenzae*-host interplay, has greatly moved forward our understanding of this human-adapted pathogen, responsible for multiple acute and chronic infections of the respiratory tract. In this way, essential genes, virulence factors, pathoadaptive traits, and multi-layer gene expression regulatory networks with both genomic and epigenomic complexity levels are being elucidated. Likewise, the unstoppable increasing whole genome sequencing information underpinning *H. influenzae* great genomic plasticity, mainly when referring to non-capsulated strains, poses major challenges to understand the genomic basis of clinically relevant phenotypes and even more, to clearly highlight potential targets of clinical interest for diagnostic, therapeutic or vaccine development. We review here how genomic, transcriptomic, proteomic and metabolomic-based approaches are great contributors to our current understanding of the interactions between *H. influenzae* and the human airways, and point possible strategies to maximize their usefulness in the context of biomedical research and clinical needs on this human-adapted bacterial pathogen.

## 1 Introduction

The Gram negative bacterium *Haemophilus influenzae* is a human-adapted pathogen, commonly associated with human disease in both children and adults. While asymptomatic colonisation begins in the upper airways, it can spread through the respiratory tract, and potentially lead to invasive infections. *H. influenzae* consists of capsulated strains (serotypes a to f), and non-capsulated strains designated as non-typeable (NTHi). The capsulated *H. influenzae* type b (Hib) was the predominating disease-causing serotype, with a high incidence of invasive Hib disease including meningitis and sepsis, until the introduction of the efficient polysaccharide capsule vaccine in the late 1980s/early 1990s, which has driven its almost complete disappearance in countries with established child immunisation programs [1]. In contrast, other *H. influenzae* serotypes and NTHi, which are not targeted by the Hib vaccine, are currently recognised as important causes of infections. In particular, NTHi is a common coloniser of the upper airways in healthy individuals, responsible for multiple acute and chronic infections of the respiratory tract, including otitis media (OM), conjunctivitis, sinusitis and lower respiratory infections in children; exacerbations of chronic obstructive pulmonary disease (COPD) and cystic fibrosis (CF) in adults; and sepsis in neonates, immunocompromised adults, and the elderly [1], [2], [3], [4], [5]. Furthermore, *H. influenzae* antimicrobial resistance presents an overall increased trend, in such a way that ampicillin-resistant *H. influenzae* is included in the WHO global priority list of bacteria for which new antibacterial agents are urgently needed [6]. Additionally, fluoroquinolone-resistant *H. influenzae* has quickly increased in recent years and spread worldwide with a variety in epidemiology [7].

**Fig. 1:**
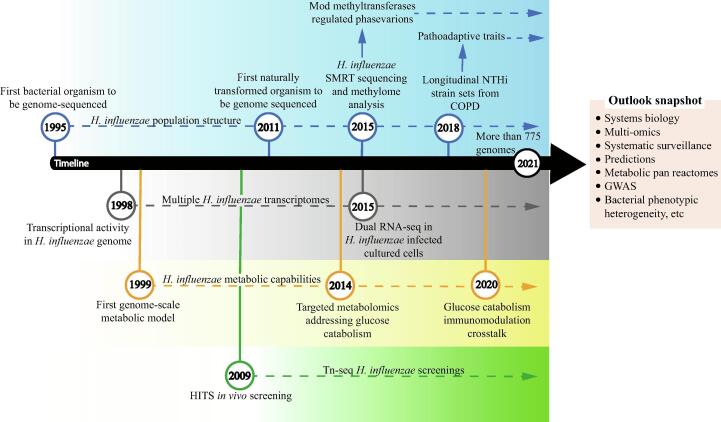
Summarized landscape of –omics contribution to our understanding of the interactions between *H. influenzae* and the human host. The timeline starts when the first *H. influenzae* complete genome was sequenced, as a major breakthrough on our understanding of the biology, diversity and evolution of bacteria. We highlight key genomic (labeled in blue), transcriptomic (labeled in grey), metabolomic (labeled in yellow), and Tn-seq screening (labeled in green)-based milestones and contributions over time. Information regarding *H. influenzae* population structure, genome-wide expression profiling, metabolic capabilities, and genetic screenings keeps growing along the entire timeline (indicated with dotted arrows). SMRT, single-molecule real-time; NTHi, nontypeable *Haemophilus influenzae*; COPD, chronic obstructive pulmonary disease; dual RNA-seq, dual RNA-sequencing; HITS, high-throughput insertion tracking by deep sequencing; Tn-seq, transposon sequencing; GWAS, genome-wide association studies. (For interpretation of the references to colour in this figure legend, the reader is referred to the web version of this article.)

The *H. influenzae* strain RdKW20 was the first free-living organism for which the complete genome sequence was established [8], the first naturally transformed organism to be genome-sequenced [9], generated the first genome-scale metabolic model [10], [11], and pioneered transposon sequencing methodologies [12]. We further review the contribution of genomic, transcriptomic, proteomic and metabolomic approaches to our understanding of the interactions between *H. influenzae* and the human airways (for a summary, see Fig. 1 and Table 1).

**Table 1:** Summary of –omics studies contributing to the *H. influenzae*-host interplay.

Type of study	Purpose-outcome of the study	References
A. *H. influenzae* bacterial whole genome sequencing	A1. Population structure of capsulated and non-capsulated (non-typeable) clinical isolates	[13], [14], [15], [16], [17], [18], [20], [21], [22], [23], [24]
	A2. Within-host pathoadaptive traits on longitudinally collected strain sets from chronic respiratory samples – genome evolution in natural settings	[32], [33], [34], [36]
	A3. Natural transformation and recombination-driven genetic variation	[9], [14], [32], [38], [39], [45], [46]
	A4. Methylome profiling	[35], [52]
	A5. Microevolution in experimental settings	[60], [61]
B. Genome-wide genetic screening	B1. HITS* based *in vivo* genetic screenings	[12], [67], [68]
	B2. Tn-seq based *in vitro* genetic screenings	[69], [70], [71], [72], [73]
C. Genome-wide gene expression profiling	C1. Bacterial transcriptomic responses to changing environmental conditions	[75], [76], [77], [78], [79], [82], [84], [87]
	C2. Bacterial transcriptomic responses to gene inactivation	[35], [53], [54], [55], [59], [80], [81], [83]
	C3. Dual RNA-seq gene expression profiling	[87]
D. Proteomic profiling	D1. Bacterial responses to changing environmental conditions	[89], [91], [92], [93]
	D2. Bacterial responses to gene inactivation	[54], [55], [81]
E. Metabolomic profiling	E1. Bacterial responses to changing environmental conditions	[99], [100], [101]
	E2. Bacterial responses to gene inactivation	[81], [100]

*HITS, high-throughput insertion tracking by deep sequencing; Tn-seq, transposon sequencing; dual RNA-seq, dual RNA-sequencing.

## 2 Constantly evolving information from *H. influenzae* whole genome sequencing

### 2.1 *H. influenzae* population structure

On a genomic basis, a whole range of multilocus sequence typing (MLST) and core genome-single nucleotide polymorphisms (core-SNP)-based studies, performed on different *H. influenzae* strain sets encompassing capsulated and non-typeable clinical isolates, commonly share that capsulated strains concentrate into a small number of serotype-specific clusters or clonal populations, while NTHi strains display great genomic diversity [13], [14], [15], [16], [17], [18]. About 779 *H. influenzae* genomes are found currently in publicly available databases (May 21st, 2021), and we expect this number to grow exponentially over time [19], making genomic information dynamic as constantly evolving data requiring regular update. In this context, *H. influenzae* competence in acquiring genomic material throughout the infection process facilitates genomic plasticity (see Section 2.3) leading to an open pan-genome which, for NTHi, was recently updated to 12,249 genes where the core genome accounts for about 7% [20]. The large accessory pan-genome confers diversity and is highly heterogeneous among strains. Many genes are phage-associated, leading to numerous isolates with distinct intact phages integrated in their genome [17], [21].

When focusing on the capsule locus, Regions I (containing the *bexABCD* operon) and III (containing the *hcsA* and *hcsB* genes), involved in exporting the capsule polysaccharide, are conserved among all six capsular serotypes, while Region II genes, required for polysaccharide synthesis, are unique to each serotype. Notably, in the frame of transition to whole genome sequencing (WGS)-based surveillance, bioinformatics tools for *in silico* rapid determination of *H. influenzae* serotypes from WGS data have been developed [13], [17]. Moreover, capsulated strains genomic closeness has been illustrated for invasive *H. influenzae* type f (Hif) and type a (Hia) isolates [22], [23]. Thus, Hif may present a pattern of unique or missing genes maybe increasing the virulence, with the presence of the *sap2* operon, *aef3* fimbriae, and genes for kanamycin nucleotidyltransferase, iron-utilization and putative YadA-like trimeric autotransporters, and the absence of an operon for *de novo* histidine biosynthesis, a *hmg* locus for lipooligosaccharide (LOS) biosynthesis and biofilm formation, and a molybdate transport system [22].

Efforts to define the structure of the NTHi population and to identify groups of related strains led to strain partitioning into six or eight groups or monophyletic clades (clades I-VI / clades I-VIII) based on the core alignment, not predicted on the basis of MLST data, and supported by the composition of the accessory genome [18], [20]. The clade I-VI classification allowed proposing clade-specific signatures regarding the exclusive presence/absence of genes encoding surface-associated proteins and LOS components [17], [18]. This sorting was further revisited on the clade I-VIII classification by applying pan-genome-wide association studies (GWAS) tools to identify genes enriched in each specific clade [20].

On the other hand, the molecular basis underpinning NTHi transition from commensal to pathogen is not clearly understood. Clade classification did not group strains of common clinical or geographical origin [18], [20], and comparative genomics, by analysing paired isogenic strains isolated from the nasopharynx (NP) and bronchoalveolar lavage (BAL) of children with chronic lung disease, did not shed light on this matter as pairs were highly genetically similar [24]. Nevertheless, and despite NTHi heterogeneity, the previously reported association between the NTHi ST14CC-PBP3IIb/A clonal group, increased clinical virulence, antimicrobial resistance and persistence over time, together with NTHi implication in clonal invasive disease, highlights that continuous surveillance and WGS in a clinical laboratory will certainly allow to rapidly address concerns of usually virulent clones or possible outbreaks, particularly in the severe immunocompromised population [25], [26], [27].

### 2.2 NTHi isolates from COPD patients: genetic signatures and pathoadaptive traits

COPD is an emerging disease worldwide, and cigarette smoking is its main risk factor. The disease is characterised by dyspnea, chronic cough, sputum production and persistent airflow limitation. COPD exacerbations are often associated to bacterial pathogens, most importantly to NTHi. A main feature of COPD is, however, a chronic inflammation of the pulmonary parenchyma that is associated with bacterial persistence, and NTHi is a frequently found bacterial species in the airways of these patients [28], [29], [30], [31]. When looking for genetic distinctions between NTHi isolates from COPD with respect to other illnesses, the distribution of core genome SNPs does not separate COPD from non-COPD strains [18], [20]. However, this distinction is possible when applying discriminant analysis of principal components to the composition of the accessory genome, further supported by pan-GWAS identification of a repertoire of unique NTHi accessory genes significantly associated with COPD, many of them with predicted roles in virulence, transmembrane transport of metal ions and nutrients, cellular respiration and maintenance of redox homeostasis [20].

Moreover, NTHi also alters its genome during persistence within the lower airways of chronic respiratory patients, as stated by the analyses of longitudinally collected NTHi strains from independent cohorts of COPD patients [32], [33], [34]. Pettigrew's group revealed frequent genetic variation due to slipped-strand mispairing in simple sequence repeats (SSR), and diversifying selection of several candidate vaccine antigens during NTHi persistence in the human airways [33]. Follow up studies on this same strain collection showed different proportions of methyltransferase-encoding *modA* alleles compared to those isolated from OM patients [35], and changes in the expression of the IgA protease variants A1, A2, B1 and B2 conferred by changes in genomes during persistent infection which, depending on the variant, could be indels or slipped-strand mispairing in mono- or heptanucleotide repeats [36]. On our part, we addressed molecular genetic changes underlying bacterial pathoadaptation by investigating genetic variants arising from within-patient evolution of NTHi in an independent cohort of COPD patients. Notably, we identified recurrent polymorphisms in several genes and provided experimental evidence for the biological significance of such convergent variation in the *ompP1-fadL* gene, whose recurrent mutations are a likely case of antagonistic pleiotropy during adaptation of NTHi to chronic lung infection associated to COPD [32].

### 2.3 WGS insights into DNA uptake, natural transformation and natural genetic variation by *H. influenzae*

Natural competence allows bacteria to respond to environmental and nutritional cues by taking up free DNA from their surroundings, gaining both nutrients and genetic information [37]. *H. influenzae* intrinsic transformable nature, self-preferences caused by the uptake machinery bias for uptake signal sequences (USS), and the high rate of homologous recombination events they can undergo, have a great impact on the evolution of the genomes of this pathogen, which seems to be higher in NTHi compared to capsulated strains [38], [39].

Such frequent genetic exchange also occurs among *Haemophilus* spp., (*H. influenzae*, *H. aegyptius* and *H. haemolyticus* belong to the cluster “*Haemophilus sensu stricto*” [40], [41]), causing frequent misidentifications of *H. influenzae* from clinical specimens when using selective culture methods, a limitation overcome by using matrix-assisted laser desorption/ionization time-of-flight (MALDI-TOF) mass spectrometry [42], [43]. On this matter, phylogeny studies may also allow not only seeking for *H. influenzae*-specific signatures of diagnostic potential such as the *fucP* locus [16], but also speciating *Haemophilus* isolates from COPD, further supporting that the heterogeneity of NTHi may indeed provide a genetic continuum between NTHi and *H. haemolyticus* [44].

When focusing on bacterial genome shaping by natural transformation, it is noticeable that the first WGS characterization of recombination events was performed by using DNA from a clinical isolate of NTHi (strain 86-028NP) to transform competent cells of a laboratory strain (strain RdKW20), leading to the first genome-wide analysis of chromosomes directly transformed with DNA from a divergent genotype [9], [45]. Such observations were independently supported by transformation of the same laboratory strain with donor DNA from a heterologous Hib strain [14]. Detailed genome-wide analyses of not only *H. influenzae* bacteria experimentally transformed with donor DNA from diverged clinical isolates, but also DNA uptake across the outer membrane of naturally competent *H. influenzae* bacteria, turned out to be extremely informative to understand the relevance and evolution of uptake sequences in the genome, and even to predict the outcome of competition between self- and non-self DNA in the respiratory tract environment [39], [45]. Moreover, transformation-based experimental evolution studies showed that transformation of single competent cells is extensive, suggesting that it could be used as a tool to map traits that vary between clinical isolates. This notion was applied to develop “transformed recombinant enrichment profiling” (TREP), in which natural transformation is used to generate complex pools of recombinants, phenotypic selection is used to enrich for specific recombinants, and deep sequencing is used to survey for the genetic variation responsible. TREP was applied to investigate the genetic architecture of intracellular epithelial invasion by *H. influenzae*, and identified specific allelic variants of the HMW1 adhesin as a key factor on this matter [46]. Connecting such *in vitro* transformation studies with the high levels of natural genetic variation found in NTHi, variation within closely related genomes from clinical strains collected from COPD respiratory samples shows numerous closely spaced variants which resemble natural transformation events seen in the laboratory, i.e. recombination tracts distinguishing the clinical isolates [32].

### 2.4 Single-molecule real-time (SMRT) sequencing contribution to *H. influenzae* phase-variable methylome analyses

Formation of C-/N-methyl-cytosine and N-methyl-adenine in bacterial genomes is a postreplicative phenomenon that occurs at specific targets, has multiple roles in bacterial physiology, and can be genome-wide deciphered by using SMRT or nanopore sequencing [47], [48], [49]. DNA methyltransferases can exist as part of restriction-modification (R-M) systems and be subject to phase variation, i.e. the random and reversible switching of gene expression due to the presence of inverted repeats (IR) or SSRs in their coding regions. Phase-variable methyltransferases control the expression of multiple genes via epigenetic mechanisms [50], [51], [52]. Such systems, called phasevarions for phase-variable regulons, were originally identified in *H. influenzae*, where the DNA methyltransferase ModA is a type III R-M system component containing a variable number of SSRs within its coding sequence [53]. ModA phase variation generates ON/OFF protein variants resulting in changes in methylation patterns, and therefore in phasevarion expression profiles and phenotypic heterogeneity. Besides phase variation, ModA also undergoes allelic variation among *H. influenzae* strains contributing to a wide range of predicted DNA methylation target sequences [50], [51], [52]. By analysing *modA* allelic variation across NTHi isolates from healthy individuals, OM and COPD patients, ModA1 to ModA21 variants have been identified, whose distribution and prevalence vary depending on the strain origin, suggesting that different *modA* alleles may provide distinct advantages in the differing human body niches and even have a marker potential [35], [54].

Several studies tackled phenotypic heterogeneity due to NTHi ModA2 phase variation, showing that (i) under alkaline conditions such as those in the middle ear during chronic disease, NTHi strains expressing ModA2 (*modA2* ON) form biofilms with greater biomass and less distinct architecture than those formed by a ModA2-deficient population (*modA2* OFF) [55]; (ii) ModA2 ON are more sensitive to oxidative stress than ModA2 OFF variants [56]. Regarding *in vivo* selection during infection of the middle ear in a chinchilla model of OM, *modA2* ON was preferentially selected compared to *mod2A* OFF [54], further supported by that the fact that a shift from OFF to ON within the middle ear results in increased disease severity [57], and that a unique host immune response may be mounted against each discrete *modA* subpopulation [58].

In addition, coupling of SMRT sequencing with gene and/or protein expression profiling allows combining knowledge of methylation specificity and gene expression changes proportional with methyltransferase phase variation. Thus, genome-wide gene expression profiling showed (i) differences when comparing RdKW20 WT and *modA* mutant strains for genes encoding, among others, surface-exposed proteins, transporters and heat-shock proteins [53]; (ii) differences when comparing R2866 WT and *modA10* mutant strains, including upregulation of membrane protein encoding genes such as *olpA2*, which is involved in host cell adhesion and invasion [59]; (iii) the effects of *modA2* phase variation, including those within biofilms by using an ON/OFF strain pair [54], [55]; (iv) the effects of *modA15* and *modA18* phase variation, by using a single ON/OFF pair of strains for each *modA* allele. In the case of the ModA15 phasevarion, the DNA binding transcriptional regulator Fis was up-regulated in ON relative to OFF, as a likely case of regulation of a regulator [35]. Conversely, proteomic analyses (i) determined relative protein abundances within the biofilms formed by each *modA2* subpopulation, with the greatest differences occurring in alkaline conditions including a significant decrease in abundance of the DNABII DNA-binding protein HU within biofilms formed by *modA2* ON compared to those formed by *modA2* OFF bacteria [55], and (ii) showed differences related to changes in the expression of outer membrane proteins when comparing *modA* ON/OFF strain pairs [54].

### 2.5 Genome-scale microevolution and genetic screening approaches shed light on NTHi adaptation and pathogenesis

Unravelling pathoadaptive genome evolution related to chronicity in the natural setting is not only thrilling but also challenging, as it requires longitudinal and preferably prospective clinical isolate sampling [32], [33]. This notion, when applied to well-suited experimental settings, also allows unravelling microevolution traits contributing to the pathogen persistence. This is the case of the mutation of the *icc* gene, encoding a 3́,5́-cyclic adenosine monophosphate phosphodiesterase, identified as a *H. influenzae* microevolution trait in response to transient nutrient limitation, which associates with increased development of intracellular bacterial communities (IBC) [60]. Likewise, when using a pre-clinical model of OM to assess NTHi pathoadaptation during sequential episodes of disease, microevolution of haemoglobin binding and LOS biosynthesis genes was observed in such OM-adapted strains, which in turn promoted increased biofilm formation, inflammation, stromal fibrosis, and an increased propensity to form IBCs [61].

On the other hand, sequencing and data analysis tools have also revolutionized genome-wide gene discovery aimed to determine gene disruptions differentially represented in a mutant population upon screening. Historical limitations by the labour involved in mapping the locations of the mutations, are overcome by using random transposition insertion mutagenesis followed by deep sequencing. This is achievable by high-throughput insertion tracking by deep sequencing (HITS), insertion sequencing (IN-seq), transposon sequencing (Tn-seq), or transposon-directed insertion sequencing (TraDIS), collectively referred to as Tn-seq [62], [63], [64], [65], [66]. *H. influenzae* pioneered genome-wide gene discovery by starring the original HITS proof of concept, developed to analyse genes required by *H. influenzae* to resist clearance from the lung in a murine pulmonary model, and identified a whole range of genes with reported or potential roles in survival during nutrient limitation, oxidative stress, and exposure to antimicrobial membrane perturbations [12]. HITS was also useful to identify *H. influenzae* genes required to colonize an influenza A virus (IAV) co-infection murine model, showing genes both exclusive or commonly shared by bacterial and IAV co-infection model systems [67], or to screen genome-wide genetic interactions to unravel epistatic interaction accounting for *H. influenzae* survival in the murine lung [68]. Tn-seq suitability has also been reported for *H. influenzae in vitro* genome-wide screening studies including the identification of (i) essential genes [69]; (ii) the carbonic anhydrase involvement in NTHi adaptation to changes in environmental CO_2_ levels and intracellular survival [70]; (iii) a role for the outer membrane protein P5 in NTHi survival to complement-mediated killing [71], and for galactose-containing oligosaccharide structures in NTHi survival to complement-dependent neutrophil-mediated killing [72]; (iv) the contribution of the *mltC* and *lppB* genes to biofilm formation [73].

## 3 Unraveling features of the *H. influenzae*-host interplay by genome-wide gene expression profiling

Since the first high-density oligonucleotide probe array containing probes representing 106 *H. influenzae* genes [74], microarray and later on RNA sequencing (RNA-seq) technologies have greatly contributed to broaden our understanding of NTHi physiology and host interplay. Please note that profiling of gene expression changes regulated by methyltransferases and their phase variation is reviewed in Section 2.4. Moreover, *H. influenzae* transits between niches within the host that differ in oxygen levels, and the ArcAB two-component regulatory system controls gene expression in response to different respiratory conditions of growth. Gene expression profiling contributed not only to identify the *H. influenzae* ArcA regulon upon anaerobic growth [75], but also to decipher changes in *H. influenzae* transcriptional responses upon changes in oxygen availability, showing that, in high oxygen, arginine uptake, arginine/aspartate metabolism, and glutamate pathways leading into the Krebs cycle are commonly up-regulated [76].

Genome-wide expression profiling also identified a putative core of genes responsive to iron and heme (FeHm) availability in several NTHi strain backgrounds including RdKW20, 10810, R2866, 86-028NP or R2846. Included in the core iron/heme modulon were genes preferentially expressed under iron/heme limitation, most of which are directly involved with iron and/or heme acquisition [77], [78], [79]. The ferric uptake regulator (Fur) regulon has also been studied, which identified the first small RNA described in any *Haemophilus* species, HrrF, overexpressed in the absence of Fur and responsive to iron levels, in turn regulating the expression of molybdate uptake, and deoxyribonucleotide and amino acid synthesis genes [80]. As mentioned above, transient nutrient limitation, in particular FeHm restriction, increases the longevity of NTHi survival *in vitro* and promotes bacterial microevolution by mutation of the *icc* gene [60]. Such adaptive trait, besides increased development of IBCs [60], also relates to increased biofilm formation and gene expression changes within the *in vitro* biofilms, some of them associated to competence and therefore likely linked to the observed increased transformation efficiency of those biofilms [81].

Regarding natural competence, changes in gene expression during competence development confirmed the existence of a competence regulon characterized by a promoter-associated competence regulatory element (CRE) closely related to the cAMP receptor protein (CRP) binding consensus, and where the essential competence gene *sxy* is induced early in competence development [82]. Such observations were further extended by differential gene expression profiling of *H. influenzae* wild-type, *crp* and *sxy* mutant strains, which also shed light on the competence-regulated toxin-antitoxin ToxTA system [83]. Moreover, analysis of global transcriptional changes has also been of use when assessing *H. influenzae* response to antibiotic pressure, showing increased *ponB* (encoding PBP1b) and *acrR* (negative regulator of AcrAB-TolC efflux pump) gene expression during heat stress, which may relate to the observed decrease in bacterial viability after incubation with imipenem at 42 °C as compared to 37 °C [84]. An effort to identify convergent molecular signatures related to lung adaptation led to comparative transcriptomic analysis of paired, isogenic NTHi strains, isolated from the NP and BAL of children with chronic lung disease, showing no convergence at the gene level, but a possible trend in terms of functional enrichment among genetically unrelated NTHi strains [24].

Although most studies have analyzed *H. influenzae* gene expression, host response to bacterial infection also needs obvious attention. Gene expression profiling identified host functions differentially expressed in NTHi-infected human type II pneumocytes, which allowed identifying a repertoire of host target candidates for pharmacological modulation [85]. In an independent study, the transcriptome of a complete episode of acute OM in mice identified sets of genes involved in regulation of immune responses, changes in epithelial and stromal cell markers, and the recruitment/function of neutrophils and macrophages [86]. Notably, a dual RNA-seq approach was undertaken for pathogen and host genome-wide expression profiling during *H. influenzae* infection of ciliated human bronchial epithelial cells. Temporal profiling of host mRNA signatures revealed significant dysregulation of the target cell cytoskeleton elicited by bacterial infection, with a profound effect on the intermediate filament network and junctional complexes. In turn, NTHi downregulated its central metabolism, and increased the expression of transporters, suggesting a change in the metabolic regime due to the availability of host substrates (see Section 4), and of stress-induced defense mechanisms, including the transport of exogenous glutathione and activation of toxin-antitoxin family components [87]. Currently lacking, *in vivo* dual RNA-seq would likely be of great help to gather a more comprehensive view of the *H. influenzae*-host interplay.

## 4 Update on *H. influenzae* protein and metabolite profiling

Proteomic studies on the *H. influenzae*-host interaction are relatively scattered. From a diagnostic perspective, *H. influenzae* species-unique peptide biomarkers discovered by tandem mass spectrometry may be a valuable tool for clinical sample proteotyping [88]. Protein profiling regulated by methyltransferase phase variation is reviewed in Section 2.4. Independently, proteomic expression profiling of *H. influenzae* grown in pooled human sputum revealed increased expression of antioxidant, stress-response proteins, and cofactor and nutrient uptake systems compared to media grown cells [89]. Definition of the protein content of NTHi lysates identified numerous unique *H. influenzae* proteins contributing to biofilm formation, immune evasion, or epithelial inflammation, classified as outer membrane/cell surface associated, metabolic, biosynthesis mediators, proteases, chaperones/DNA binding proteins, transporters, reductases or hydrolases [90]. Proteomic profiling was also used to demonstrate differential expression of the NTHi biofilm to planktonic samples, where ArcA showed a high level of downregulation in the biofilm [91], and whose inactivation led to ArcA-regulated proteomic changes, some of them maybe involved in serum susceptibility [92]. Proteomic profiling was also useful to prove that the two bacterial populations released from a NTHi biofilm by using antibodies directed against the type IV pilus or, alternatively, against a DNABII DNA-binding protein, are different not only from planktonically grown NTHi, but also from each other despite genetic identity. Even more, each newly released population had a distinct, increased susceptibility to antibiotic killing compared to planktonic bacteria, highlighting new clues for anti-biofilm therapeutics [93].

From the host side, proteomic and metabolomic signatures of NTHi-induced acute OM in a chinchilla model were delineated in infected middle ear tissue lysates, revealing that establishment of disease coincides with actin morphogenesis, suppression of inflammatory mediators, and bacterial aerobic respiration. As a first step toward identification of clinically meaningful metabolic biomarkers, decreased biogenic amines and sphingomyelin molecules, increased taurine, glutamine and ornithine were observed upon infection [94].

Elucidating the metabolic determinants in the lung during respiratory infection is likely to be key to develop therapeutics modulating symptom and/or disease severity [95]. Lung metabolomics have not been performed on the *H. influenzae*-human airways interplay, but currently available insights on bacterial metabolism may guide us when assessing this infectious process. The *H. influenzae* RdKW20 strain drove the first genome-scale metabolic model. Besides assessing basic structural features of the *H. influenzae* metabolic network, such model facilitated addressing minimal substrates requirements for the network to allow biomass production. Minimal requirements included fructose, a likely preferred carbon source for which a phosphotransferase system (PTS) exists in *H. influenzae* [96], [97], but fructose could also be replaced by other carbon sources including glucose [10], [11]. *H. influenzae* possesses complete glycolysis and pentose phosphate pathways for glucose catabolism, lacks most enzymes of the oxidative branch of the Krebs cycle, and holds a respiratory chain with several dehydrogenases transferring electrons into the menaquinone pool, and terminal reductases transferring the electrons to a variety of electron acceptors, altogether driving a so-called glucose respiration-assisted fermentation, where acetate is the main end-product under aerobic growth. When oxygen is limiting, acetate is still produced but other products arise including formate and succinate, at variable rates depending on the tested strains [98], [99], [100], [101]. End-product excretion profiling is informative not only from the bacterial perspective, but also due to their possible immunomodulatory roles. Although lung nutritional immunity keeps low glucose levels in the airway surface liquid of healthy individuals, respiratory disease generates a glucose-rich niche favorable for pathogens able to use glucose as carbon source. As this being the case for *H. influenzae*, glucose catabolism enhances bacterial growth, but also promotes the release of end-products acting as pro-inflammatory metabolites, more specifically acetate, which may contribute to lung colonization and inflammation in chronic respiratory patients [100]. Besides the above described genomic flexibility (see Section 2), *H. influenzae* metabolic versatility among strains has also been reported, in terms of substrate utilization, histidine synthesis or urease activity, suggesting metabolic adaptive traits favoring differential access to throat, ear, lower airways or blood niches [101], [102], [103], [104].

Finally, and considering that *H. influenzae* metabolic machinery adapts to the host-imposed milieu, transcriptome signatures upon cell infection showed that the genes encoding the bacterial biosynthesis pathways of carbohydrates, lipids, amino acids, nucleotides, and energy metabolism were mostly downregulated, although the shikimate pathway for precursor chorismate production and the consequent tryptophan biosynthesis pathway was upregulated after initial bacterial-cell contact [87]. Increased tryptophan biosynthetic pathway was also observed in biofilms formed by strain variants adapted to transient FeHm restriction where, moreover, amino acids and enzymes associated with central metabolism, and metabolites associated with the urea cycle were also increased [81]. Linked to the metabolic machinery, attention should also be paid to bacterial metabolite transport systems. In fact, lactate, arginine, tryptophan, cysteine/glutathione, thiamine, thiamine pyrophosphate, iron and polyamines uptake and transport systems were highly expressed during *H. influenzae* cell infection [87]. Likewise, increased production of serine, sialic acid and tryptophan transporters was observed in biofilms formed by strain variants adapted to transient FeHm restriction [81], together suggesting that changes in central metabolism combined with increased nutrient stores may help counteracting nutritional immunity at the colonizing niches.

## 5 Future outlook

The last twenty five years, since the *H. influenzae* RdKW20 strain was genome sequenced, have gone a long way, greatly propelled by the unstoppable –omics technologies. As a human-adapted pathogen, integrative systems biology approaches to the interactions between *H. influenzae* and the human airways, are instrumental in the discovery of specific bacterial recognition, host signal transduction or immune tolerance, together with multi-omics approaches to decipher the genetic, immunologic, (post)transcriptional, (post)translational, and metabolic mechanisms underlying the outcome of such interactions. Those approaches will be enormously enriched by bacterial single-cell resolution methodologies such as microfluidics, or single-cell sorting, microscopy and genome/RNA sequencing, to allow overlooking the remarkable bacterial phenotypic heterogeneity, and shed light on complex phenotypes such as antibiotic persistence or metabolic specialization. Guidance of such integrative work by the human specificity of the infection will provide meaningful information on *H. influenzae* patho-adaptive evolution within the human host. Furthermore, we are collecting a wealth of genomic and epigenomic data, and this will continue to grow with the introduction of routine sequencing for disease surveillance. These data will be of great use to predict how changes in genome sequence lead to different host-pathogen interaction outcomes by applying machine learning approaches, or to build genome-scale metabolic network reconstructions assessing core and pan reactomes. Also, combination of deep bacterial phenotyping and pan-GWAS methodologies will allow not only predicting bacterial genotype-phenotype associations, but also deciphering genetic determinants of resistance to antimicrobials, virulence or niche adaptation. Altogether, therapeutic/vaccination target and biomarker discovery will be moved forward, to be eventually translated to the clinical settings and reach end users.

## Funding

N.L.-L. is funded by a PhD studentship from Regional Navarra Govern, Spain, reference 0011-1408-2017-000000. C.G.-C. is funded by a PhD studentship from Agencia Española de Investigación (AEI), Spain, reference PRE2019-088382. This work has been funded by grants from MINECO RTI2018-096369-B-I00, 875/2019 from SEPAR, and PC150-151–152 from Gobierno de Navarra to J.G. CIBER is an initiative from Instituto de Salud Carlos III (ISCIII), Madrid, Spain.

## Author contributions

Conceptualization: J.G.; writing (original draft preparation): N.L.-L. C.G.-C. and J.G.; writing (review and editing): all authors; supervision: J.G.; funding acquisition: J.G.

## Declaration of Competing Interest

The authors declare that they have no known competing financial interests or personal relationships that could have appeared to influence the work reported in this paper.
